# Supplementary material for: Patterns of Intron Gain and Loss in Fungi
Source: PLoS Biol. 2004 Nov 30;2(12):e422. doi: 10.1371/journal.pbio.0020422 (PMC532390; doi:10.1371/journal.pbio.0020422)
Supplement: Table S1 — Also available at http://genes.mit.edu/NielsenEtAl/. (4.3 MB ZIP). [file pbio.0020422.st001.zip › NielsenEtAl/html/401.html]

AN4578.1.NCU02360.1.MG03534.1.FG07966.1


```
 CLUSTAL W (1.82) Multiple Sequence Alignments - Introns Inserted


Sequence 1: MG03534.1	393 aa
Sequence 2: FG07966.1	358 aa
Sequence 3: NCU02360.1	386 aa
Sequence 4: AN4578.1	362 aa
Alignment Length: 401 aa
Number Identitical Residues: 150 aa
Alignment Score (without introns) 8516


MG03534.1 	-MPAEPVQIDK------EKIKKAFDVSNFDFNAVLRGMQLTLVGA1HRALQNPAIFTSEH
NCU02360.1	MATADRLERRQSFDVVAEQVKRKFDMSHFDLNAIIRGAQLTLVGA1QRALQNPGIFTSRH
FG07966.1 	MSSNENMSIDKDK--LKEKLEQKLDVSHFDPNTVIRGAQLTLVGA1HRALQNPALFTTDH
AN4578.1  	-------------------------MSSFEPNAVIRGAQLTVVGT1VRALINPRLFKYDH
          	                         :* *: *:::** ***:**:  *** ** :*.  *

MG03534.1 	YRQALYAVGAGIAIRLAIAIP0ILGIKVLLWVLSFVFRMDGVTWDDKLVDGLNFVGEYVL
NCU02360.1	YKQAAIAVAAGILIRLLISIP0IIGIRVLLWFLSFGIDLRNATWDDKLVGGLGFVEEHVL
FG07966.1 	YRQAAIAVVAGLAIRLVISVP0IVGIKLLLWLISFFVSLNAVTWDDTLVNGLDFVAEYVL
AN4578.1  	FRQAALAIGIGIIIQLIIQIP0IISLRLLIWITSWIVNLEDATWDDALLNSLDFLNKSVL
          	::**  *:  *: *:* * :* *:.:::*:*. *: . :  .**** *:..*.*: : **

MG03534.1 	QVPLFLMSMMRLISPTLDNL2FMDSLRWVDLTYVQKHKR-DNPDELRDMYYPNMRMYKQR
NCU02360.1	QAPLFFMSLMRYVTPTLDDL2FMDSIRWVDTTYVNKHKHDEDPSKLRPMYYPNLRQYSVR
FG07966.1 	QVPFFLMALMRYVVPTLDNL2N------------------EKPSELRDMYYPNLKMYRPT
AN4578.1  	QVPFLLMTLLRYLVPTLDEI2FMDSLNWVDTTYVHKHKT-DDPNTLRAMYYPNLVQYPTA
          	*.*:::*:::* : ****::   .:    . :   . .  :.*. ** *****:  *   

MG03534.1 	DGSTNSTSTAEASSMFIWRFARKAFISLAMFALSYVPVIGRFVLPAASFYTFKKAVGLGP
NCU02360.1	DGTTNSTSTAENISMFIYRFARKGAISLAVFAASYLPYIGRFVLPAASFWTFNRAVGLGP
FG07966.1 	DGSTHSESTAQAVSMFLYRFLRKGGISLAVFALSYTPYIGRFVLPAASFYTFNNAVGLGP
AN4578.1  	NGKGESKPVVKKLMAFLKRYGRKVGMLLGIYLVSLVPVVGRFVMPAASFYTFHRAVGTTP
          	:*. .* ...:    *: *: **  : *.::  *  * :****:*****:**:.***  *

MG03534.1 	ASVIFGTGVLMPRKYLIIFLQTYFSSRNLMREL~LEPYFSRIHMTKEQKKRWFRTREGIL
NCU02360.1	ASVIFGTGILLPRRYLVIFLQSYFSSRSLVREL~LEPYFARIRFTSEQKRKWFRSREGLL
FG07966.1 	ASVIFGTGIFLPRKYLVIFLQSYFSSRSLMREL~LEPYFARVHFTKQQKRNWFRSREGVL
AN4578.1  	AAVIFGTGILLPKRYLVTFLHSYFASRSLMREL0LEPYFSRIPFTSEQKSRWFKDREGVL
          	*:******:::*::**: **::**:**.*:*** *****:*: :*.:** .**: ***:*

MG03534.1 	FGFGIGFYILL0RVPLLGVLIYGIAEA~STAYLITKITDPPPPPSELAAYVESQKVWKNK
NCU02360.1	FGFGIGFYILL~RVPLLGVLIYGIAEA0SAAYLITKVSDPPPPPSESSGFAESQLEWRNK
FG07966.1 	FGFGLGFYVLV~KIPLVGVLVYGIAEA~STAYLITKITDPPPPPQQMNEFTQGQQNWSNK
AN4578.1  	FGFAFAFTFVL~KIPIIGVLMYGIAEA~STAYLITKITDPPPSPAESEGFAETQVTWKNK
          	***.:.* .:: ::*::***:****** *:******::****.* :   :.: *  * **

MG03534.1 	HEFLSLSLGDLDALQMNKSVTASSNKPPPYSEQAPGTGNSTGMASHG
NCU02360.1	QKFLNLSLANMDSIHDKPPPYSIHD-PNPKTEL--------------
FG07966.1 	HEFLNLSLANIDSVHTEDSLKKAK-----------------------
AN4578.1  	HDFLRLSLDNIDKLNVAAHEQPSPKPATPGRKFA-------------
          	:.** *** ::* ::         . . .  . :
```
